# Management and outcomes of adolescent and young adult sarcoma patients: results from the French nationwide database NETSARC

**DOI:** 10.1186/s12885-023-10556-4

**Published:** 2023-01-20

**Authors:** Pierre Kubicek, Axel Le Cesne, Cyril Lervat, Maud Toulmonde, Christine Chevreau, Florence Duffaud, Louis-Romée Le Nail, Magali Morelle, Nathalie Gaspar, Cécile Vérité, Marie-Pierre Castex, Nicolas Penel, Esma Saada, Sylvain Causeret, François Bertucci, Christophe Perrin, Emmanuelle Bompas, Daniel Orbach, Valérie Laurence, Sophie Piperno-Neumann, Philippe Anract, Maria Rios, Jean-Claude Gentet, Éric Mascard, Stéphanie Pannier, Pascale Blouin, Sébastien Carrère, Loïc Chaigneau, Pauline Soibinet-Oudot, Nadège Corradini, Pascaline Boudou-Rouquette, Jean-Christophe Ruzic, Valérie Lebrun-Ly, Pascale Dubray-Longeras, Sharmini Varatharajah, Céleste Lebbe, Mickaël Ropars, Jean-Emmanuel Kurtz, Cécile Guillemet, Jean-Pierre Lotz, Juliane Berchoud, Grégory Cherrier, Françoise Ducimetière, Claire Chemin, Antoine Italiano, Charles Honoré, Emmanuel Desandes, Jean-Yves Blay, François Gouin, Perrine Marec-Bérard

**Affiliations:** 1 Department of Medical Oncology, Institut de Cancérologie de l’Ouest, Angers, France; 2 Centre Léon Bérard, Lyon, France; 3 Gustave Roussy, Villejuif, France; 4 Centre Oscar Lambret, Lille, France; 5 Institut Bergonié, Bordeaux, France; 6 Institut Claudius Régaud IUCT Toulouse, Toulouse, France; 7 CHU Timone, Marseille, France; 8 CHU Tours, Tours, France; 9 CHU Toulouse, Toulouse, France; 10 Centre Antoine Lacassagne, Nice, France; 11 Centre Georges François Leclerc, Dijon, France; 12 Institut Paoli-Calmettes, Marseille, France; 13 Centre Eugène Marquis, Rennes, France; 14 Institut de Cancérologie de l’Ouest, Nantes, France; 15 SIREDO Oncology Center (Care, Innovation and Research for Children and AYA with Cancer), PSL Research University, Institut Curie, Paris, France; 16 Hôpital Cochin, Paris, France; 17 Institut de Cancérologie de Lorraine, Nancy, France; 18 Hôpital Necker, Paris, France; 19 Centre Val d’Aurelle ICM, Montpellier, France; 20 CHU Besançon, Besançon, France; 21 Institut Jean Godinot, Reims, France; 22 Institute of Pediatric Hematology and Oncology, Lyon, France; 23 CHU La Réunion Mayotte, Saint-Pierre, France; 24 CHU Limoges, Limoges, France; 25 Centre Jean Perrin, Clermont-Ferrand, France; 26 Centre François Baclesse, Caen, France; 27 Hôpital Saint-Louis, Paris, France; 28 CHU Rennes, Rennes, France; 29 Institut de Cancérologie Strasbourg-Europe ICANS, Strasbourg, France; 30 Centre Henri Becquerel, Rouen, France; 31 Hôpital Tenon, Paris, France; 32 CHU Nantes, Nantes, France; 33 CHRU Nancy, Centre de Recherche en Epidémiologie et en Statistique Sorbonne-Paris Cité (CRESS), UMR 1153, INSERM, Université de Paris-Descartes, Paris, France

**Keywords:** Adolescents and young adults, AYAs, Sarcoma, Management, Multidisciplinary tumor board, Reference centers, Survival, NETSARC

## Abstract

**Background:**

The initial management of patients with sarcoma is a critical issue. We used the nationwide *French National Cancer Institute*-funded prospective sarcoma database NETSARC to report the management and oncologic outcomes in adolescents and young adults (AYAs) patients with sarcoma at the national level.

**Patients and methods:**

NETSARC database gathers regularly monitored and updated data from patients with sarcoma. NETSARC was queried for patients (15–30 years) with sarcoma diagnosed from 2010 to 2017 for whom tumor resection had been performed. We reported management, locoregional recurrence-free survival (LRFS), progression-free survival (PFS), and overall survival (OS) in AYA treated in French reference sarcoma centers (RSC) and outside RSC (non-RSC) and conducted multivariable survival analyses adjusted for classical prognostic factors.

**Results:**

Among 3,227 patients aged 15–30 years with sarcoma diagnosed between 2010 and 2017, the study included 2,227 patients with surgery data available, among whom 1,290 AYAs had been operated in RSC, and 937 AYAs in non-RSC. Significant differences in compliance to guidelines were observed including pre-treatment biopsy (RSC: 85.9%; non-RSC 48.1%), pre-treatment imaging (RSC: 86.8%; non-RSC: 56.5%) and R0 margins (RSC 57.6%; non-RSC: 20.2%) (*p* < 0.001). 3y-OS rates were 81.1% (95%CI 78.3–83.6) in AYA in RSC and 82.7% (95%CI 79.4–85.5) in AYA in non-RSC, respectively. Whereas no significant differences in OS was observed in AYAs treated in RSC and in non-RSC, LRFS and PFS were improved in AYAs treated in RSC compared to AYAs treated in non-RSC (Hazard Ratios (HR): 0.58 and 0.83, respectively).

**Conclusions:**

This study highlights the importance for AYA patients with sarcoma to be managed in national sarcoma reference centers involving multidisciplinary medical teams with paediatric and adult oncologists.

## Background

Sarcomas are a group of rare, highly heterogenous connective tissue cancers with more than 70 histotypes identified and a wide array of clinical presentations [1]. Estimated incidence in Europe for all age groups is 5.6/100,000 inhabitants per year, and includes soft tissue sarcomas (STS, 84%) and bone sarcomas (16%) [2, 3]. However, in adolescents and young adults (AYA), defined in France as patients aged from 15 to 24 years, more AYA patients present with bone sarcomas (standardized incidence rate of 14.6 per million), and conversely less AYA patients had STS (12.6 per million) [4].

Clinical practice guidelines for sarcoma patients recommend dedicated management involving multidisciplinary teams [5–7]. In 2009, the *French National Cancer Institute* INCa funded the national clinical sarcoma reference network NETSARC gathering 26 reference sarcoma centers to generate a nationwide institutional sarcoma data collection and improve the outcome of sarcoma patients. NETSARC highlighted that a specialized multidisciplinary tumor board (MDTB) presentation before treatment was significantly associated with a better relapse-free survival [8], and reported a reduced risk of local relapse, progression, and death in patients operated in a NETSARC reference sarcoma centers (RSC) compared to patients operated outside NETSARC centers (non-RSC), regardless of their age (Hazard Ratios (HR): 0.83 and 0.68, respectively) [9].

According to the French healthcare system, any patient < 18 years should be managed in expert centers in order to facilitate improved survival, opportunities for clinical trial inclusion, and ensure access to high-quality pluridisciplinary care [10–13]. Real-life situations revealed that the management of AYA patients with sarcoma still remains heterogeneous in France as in many other European countries, and about one third of these young patients are currently treated outside cancer centers (15–19 years: 22%; 20–24 years: 36%), and usually managed in adult departments (15–19 years: 61%; 20–24 years: 89%). Unfortunately only 85% of the medical files of AYAs in the 15–19 year and 20–24 year age ranges are currently reviewed by a MDTB before treatment, which may result in missed opportunities for clinical study inclusions. In addition, young adults, who are more frequently treated in non-expert centers compared to adolescents, are less likely to be enrolled in clinical trials (18–25 years: 16.8%; 15–18 years: 39.5%) [14], and may consequently miss access to innovative treatments. In parallel, a reduced compliance to international guidelines has been observed in patients aged 15–30 years, managed in non-RSC, with absence of pre-treatment biopsy, absence of pre-treatment imaging and failure of macroscopically complete resection. Less respect to compliance and standards was reported to associate with a decreased 10-month relapse-free survival in patients treated in non-RSC (85%) compared with those treated in RSC (93.9%) [15, 16]. Progression-free survival and overall survival are not yet available.

This retrospective study queried the nationwide French database NETSARC for patients 15–30 years with sarcoma diagnosed between 2010 and 2017, and compared patient management and outcome in RSC and in non-RSC.

## Patients and methods

### The NETSARC database

In 2009, the French National Cancer Institute (INCa) funded the clinical network NETSARC including 26 French RSC and linked to a dedicated network for expert pathology diagnosis in sarcoma (RRePS) involving 19 pathology reference centers in charge of a second pathological review for each suspected case, in order to improve the outcome of sarcoma patients. In practice, any file from a patient with suspicion of sarcoma should be reviewed by the MDTB. Patient files are presented at any time, before any diagnostic procedure, before initial biopsy, before primary surgery, after primary surgery, at relapse, and/or for eligibility for clinical trial. The current NETSARC + database (netsarc.org) has gathered all sarcoma cases presented to the MDTB from Jan 1st, 2010 and includes data on diagnosis, therapeutic management, and clinical outcomes in terms of relapse and survival [17]. From 2016, data regarding neoadjuvant treatments were added to the database. However, specific information such as use of systemic anticancer drugs, radiotherapy, or combination thereof was not collected.

This study quiered the NETSARC + database for patients with sarcoma diagnosed from 2010 to 2017, and a limited set of anonymized data of patients aged 15–30 years with sarcoma and who have been operated, was used to describe patient and tumor characteristics, quality of surgery, relapse, and survival and to compare patient management in RSC centers and non-RSC centers [8]. The *French Sarcoma Group-Groupe d’Etude des Tumeurs Osseuses* (GSF-GETO) validated this research project on Jan 18, 2020. Patients received information sheets (https://expertisesarcome.org/espace-patients/adultes/), and non-opposition procedure applied.

Data collection included patient demographics, disease status (local or metastatic) and tumor characteristics. The wider tumor diameter defined tumor size. Soft-tissue sarcoma also included viscera localisation. The *National Federation of Cancer Centres* (FNCLCC, Unicancer) specified 4 categories for histological grade: grade 1, 2, 3, and ungraded tumors. Sarcomas without grade resulted from histology grading failure or lack of critical elements to complete diagnosis, as determined by experts. The quality of surgical resection used the definition of the *Union Internationale Contre le Cancer* (UICC) [17], and margin status was issued from pathology and surgery reports when available: R0 referred to clear margins – in the present study R0 margins qualified *monobloc* resection and clear margins specified in pathological report; R1 margins referred to (possible) microscopic residual disease, with visible tumor cells on resection margins (positive microscopic margins) – in the present study R1 margins indicated margins not confirmed as R0 or R2. R2 resulted from fragmented resections, or operative/pathological reports suggesting or notifying macroscopic residual tumor and/or fragmented resection. Categorization for treatment centers were performed according to the affiliation of the surgeon in charge of the first surgery. A surgeon referenced in the NETSARC network led to consider the patient as treated in French RSC (https://netsarc.sarcomabcb.org). Conversely, a surgeon not referenced in the NETSARC network led to consider the patient as being treated in French non-RSC. Because of surgeon affiliation requirement for categorisation, patients with desmoid tumors who rarely required surgery, and patients with no surgery, or with no information on surgery were excluded.

Data from patient diagnosed from 2010 and not later than 2017 were selected to ensure at least 3-years of follow-up. The data cut-off for data analysis was June 26, 2020.

### Statistical analysis

Categorical data were expressed with frequencies and percentages, and continuous data with median and interquartile range. Comparisons were performed with chi-square for categorical variables and Mann-Whitney test (MW) for continuous data.

The diagnostic date was the date of pathological diagnosis (biopsy or first surgery). Locoregional recurrence free survival (LRFS) was computed from the diagnosis date to the date of first locoregional progression, or censored at last follow-up. Competing events to LRFS were estimated using a competing risk approach. Progression free survival (PFS) was defined as the time from the date of diagnosis to the date of first local or metastatic progression or death, whichever occurred first, or censored at last follow-up. Overall survival (OS) was defined as the time from the date of diagnosis to the date of death due to any cause, or censored at last follow-up. PFS and OS were estimated using the Kaplan-Meier method. Survival distributions between the 2 groups were compared using a log–rank test and the multivariable analysis used a Cox proportional hazard model. The cumulative incidence function and nonparametric Gray’s test were used to estimate and compare cumulative incidence function between groups and a Fine-Gray model was used for the multivariable analysis [18, 19]. Univariable analyses for LRFS, PFS and OS included the classical prognostic factors for sarcoma defined as age, gender, metastatic status at diagnosis, tumor grade, tumor size, tumor site, tumor localisation (lower limb), and management in RSC or in non-RSC, and these variables were used for adjustment in multivariable analyses. Statistical significance was set at p < 0.05. All statistical analyses were performed using SAS software (version 9.4, SAS Institute Inc., Cary, RSC, USA).

## Results

### Patient population

**Fig. 1:**
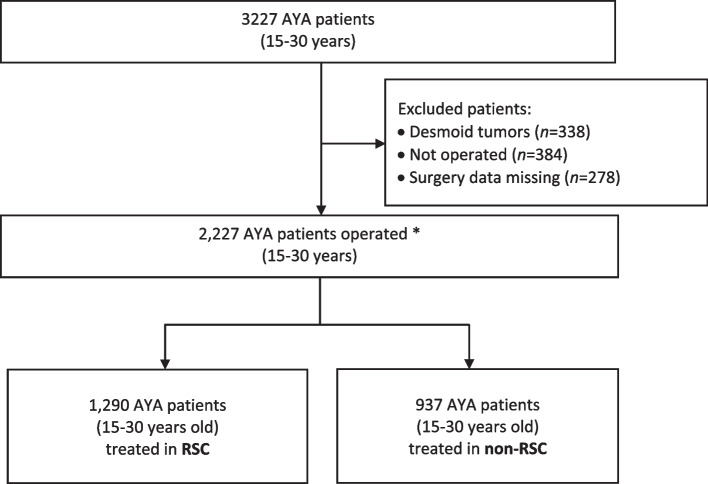
Flowchart. *Center categorisation was based on first surgeon affiliation, categorisation therefore excluded patients with desmoid tumors who rarely require resection, patients not operated, patients with surgery data missing

The NETSARC database included 3227 French patients aged between 15 and 30 years with sarcoma diagnosed between 2010 and 2017. The study excluded patients with desmoid tumors who rarely require surgery (*n* = 338) [20], patients with no surgery (*n* = 384) or patients with no information on surgery (*n* = 278). In the 2227 patients operated, 1290 patients were treated in RSC, and 937 AYAs were managed in non-reference centers (Fig. 1).

### Patient characteristics

The characteristics of AYA patients are presented in Table 1.

**Table 1:** Patient characteristics

	AYA in RSC (*n* = 1,290)	AYA in non-RSC (*n* = 937)	*p*-value
**Age (years)**	22 (18–27)	24 (20–28)	<0.001
[15–18]	338 (26%)	181 (19%)	<0.001
[18–25]	539 (42%)	390 (42%)	
[25–30]	413 (32%)	366 (39%)	
**Gender, Male**	721 (56%)	460 (49%)	0.002
***De novo*** **metastatic disease**	149 (12%)	89 (10%)	0.172
**Site of tumor**^a^			<0.001
Bone sarcoma	761 (59%)	213 (23%)	
Soft tissue sarcoma	528 (41%)	724 (77%)	
**Localisation**			
Lower limbs tumors ^a^	633 (49%)	244 (26%)	< 0.001
**Tumor size (mm)**^b^	72 (47–110)	50 (26–80)	< 0.001
**Grade of tumor**			< 0.001
Grade 1	79 (6%)	50 (6%)	
Grade 2	92 (7%)	84 (10%)	
Grade 3 ^c^	681 (54%)	371 (42%)	
Non gradable tumor	401 (32%)	368 (42%)	

Data are n (%) or median (interquartile range). Missing data: ^a^*n* = 1; ^b^*n* = 374; ^c^*n* = 110

AYAs with sarcoma treated in RSC were younger (*p* < 0.001), presented with worse prognosis, had a majority of grade 3 (54% *versus* 42%; *p* < 0.001) and larger tumor size (72 mm *versus* 50 mm; *p* < 0.001) compared with AYAs in non-RSC (Table 1). AYAs in RSC also were more likely to have bone sarcoma (59% *versus* 23%; *p* < 0.001) and lower limb tumors (49% *versus* 26%; *p* < 0.001).

### Compliance to guidelines

Compliance to guidelines in pre-treatment management significantly differs in AYAs in RSC, and AYAs in non-RSC: pre-treatment biopsies were respectively performed in 86%, and 48%, and pre-treatment imaging in 87% and 57%. Neoadjuvant therapy was significantly more reported in AYA patients in RSC (57%), than in AYAs managed in non-RSC (14%) (*p* < 0.001) (Table 1). Indeed, a majority of AYA patients with sarcoma in non-RSC had no biopsy before surgery and had identical dates for diagnosis and surgical resection (68%) (Table 1).

### Quality of surgery and reoperations

More rigorous applications of international surgery guidelines were reported in AYAs treated in RSC compared with AYAs in non-RSC, and rates of resections with R0 margins were 63%, and 22%, respectively (*p* < 0.001) (Table 2). A significantly higher rate of incomplete resections was reported in patients resected in non-RSC, with more R1 (30%) and R2 (20%) resections compared with patients treated in RSC (*p* < 0.001). Reoperations of AYAs with incomplete surgery after initial resection in non-RSC frequently occurred in the patients with initial R1 margins (56%), and in the patients with initial R2 margins (64%). AYAs initially treated in non-RSC were mostly reoperated in RSC (2d surgery, RSC: 48%; non-RSC: 31%; *p* < 0.001) (Table 2).

**Table 2:** Quality of the first surgery and second resection in AYA patients treated in RSC and non-RSC.

	Patients with first surgery*		
	**AYA in RSC (** ***n*** ** = 1,290)**	**AYA in non-RSC (** ***n*** ** = 937)**	Comparison of AYA in RSC and in non-RSC (*p*-value)
**Patient management**			
Pre-treatment biopsy ^a^	1102 (86%)	447 (48%)	< 0.001
Pre-treatment imaging	1120 (87%)	529 (57%)	< 0.001
Neoadjuvant treatment ^b^	486 (57%)	81 (14%)	< 0.001
**Diagnosis and surgery with identical dates**	226 (18%)	634 (68%)	< 0.001
**First surgery**^**c**^			0.120
Curetage	15 (1%)	5 (0%)	
Tumor resection	1275 (99%)	932 (99%)	
Quality of first surgery^*^			< 0.001
No margin	56 (4%)	26 (3%)	
R0 margins	735 (58%)	188 (20%)	
R1 margins	225 (18%)	254 (27%)	
R2 margins	48 (4%)	170 (18%)	
Unknown margins	211 (17%)	294 (31%)	
**Patients reoperated (second resection)**^**c**^	79 (9%)	271 (40%)	< 0.001
Reoperation in patients with initial R1 margins ^d^	43 (24%)	113 (56%)	< 0.001
Reoperation in patients with initial R2 margins ^e^	15 (44%)	94 (64%)	0.029
**Hospital/cancer center for second resection**			< 0.001
RSC	55 (70%)	131 (48%)	
non-RSC	5 (6%)	85 (31%)	
Unknown	19 (24%)	55 (20%)	

*excluding curetage

Diagnosis and surgery with identical dates also described as *“whoops surgery”.* “No margin” qualification issued from pathology description. “Unknown margins” indicates that margin status was not mentioned in pathology report. Missing data: ^a^ *n* = 14; ^b^*n* = 774; ^c^*n* = 642 ^d^*n* = 100; ^e^*n* = 38

### Outcomes (LRFS, PFS, OS)

The median follow-up of the total population was 39 (0.2-119.7) months.

#### LRFS

**Fig. 2:**
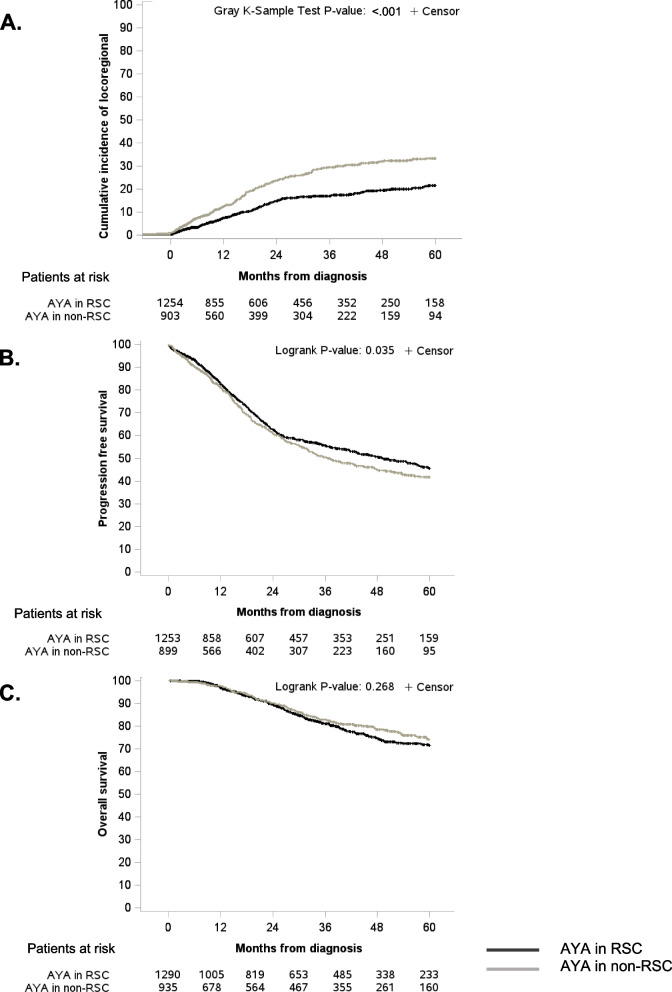
Cumulative incidence of locoregional progression (**A**) and Kaplan-Meier curves for Progression Free Survival (PFS) (**B**) and for Overall Survival (OS) (**C**). RSC : Reference Sarcoma Centers

The 3y-LRFS rates for AYA in RSC and AYA in non-RSC were 83.2% (95%CI 80.4–85.5), and 71.3% (95%CI 68.0-74.8), respectively. The cumulative incidence for locoregional progression is shown in Fig. 2A.

In univariable analysis, LRFS was significantly better in AYAs in RSC (HR 0.58, 95%CI 0.47–0.70) compared to AYAs in non-RSC (*p* < 0.001). After adjustment, the multivariable analysis still showed better LRFS patients in RSC: HR 0.58 (0.46–0.73) compared to patients in non-RSC. LRFS was reduced for ungraded tumors (HR 1.82, 95%CI 1.31–2.53). LRFS was increased for lower limb tumors (HR 0.73, 95%CI 0.59–0.92) (Table 3).

**Table 3:** Univariable and multivariable analyses for Overall Survival (OS), Progression Free Survival (PFS) and Locoregional Recurrence-Free Survival (LRFS)

	Locoregional Recurrence Free Survival		Progression Free Survival		Overall Survival	
	Unadjusted HR	Adjusted HR	Unadjusted HR	Adjusted HR	Unadjusted HR	Adjusted HR
**15–18 aged** (ref: >18years)	0.80 (0.63–1.01); 0.065	0.86 (0.67–1.10); 0.226	1.00 (0.86–1.17); 0.968	0.85 (0.72–1.01); 0.063	0.98 (0.78–1.24); 0.862	0.76 (0.60–0.98); 0.033
**Gender** male (ref: female)	0.87 (0.72–1.06); 0.169	0.91 (0.74–1.11); 0.354	1.16 (1.01–1.32); 0.034	1.05 (0.91–1.22); 0.476	1.33 (1.08–1.63); 0.007	1.20 (0.96–1.49); 0.110
***de novo*** **metastatic disease** (ref: no)	---	---	2.59 (2.18–3.07); <0.001	2.20 (1.83–2.64); <0.001	3.75 (2.99–4.68); <0.001	2.90 (2.29–3.67); <0.001
**Tumor size** ≤ 10 cm or not documented (ref:>10 cm)	1.14 (0.88–1.46); 0.292	0.94 (0.72–1.22); 0.632	2.59 (2.18–3.07); <0.001	0.79 (0.67–0.93); 0.006	0.66 (0.53–0.83); <0.001	0.82 (0.64–1.04); 0.102
**Lower limb** (ref: other)	0.67 (0.540.82); <0.001	0.73 (0.59–0.92); 0.008	0.72 (0.63–0.83); <0.001	0.76 (0.66–0.89); <0.001	0.59 (0.48–0.74); <0.001	0.60 (0.47–0.76); <0.001
**Site of tumor** Bone (ref: Soft tissue)	0.97 (0.79–1.17); 0.736	1.43 (1.13–1.81); 0.003	1.05 (0.92–1.20); 0.503	1.07 (0.91–1.25); 0.417	0.98 (0.80–1.20); 0.842	0.87 (0.69–1.11); 0.259
**Tumor grade** Grade 3 (ref: grades 1–2)	1.04 (0.75–1.44); 0.823	1.02 (0.73–1.43); 0.910	2.69 (2.09–3.46); <0.001	2.20 (1.76–2.98); <0.001	3.85 (2.51–5.88); <0.001	3.27 (2.11–5.05); <0.001
**Ungraded tumor** (ref: grades 1–2)	1.99 (1.44–2.77); <0.001	1.82 (1.31–2.53); <0.001	1.97 (1.49–2.59); <0.001	1.88 (1.42–2.49); <0.001	1.05 (0.63–1.76); 0.847	1.10 (0.65–1.86); 0.711
**AYA in RSC** (ref: AYA in non-RSC)	0.58 (0.47;0.70); <0.001	0.58 (0.46–0.73); <0.001	0.86 (0.76–0.99); 0.035	0.83 (0.71–0.97); 0.021	1.12 (0.91–1.39); 0.269	1.10 (0.87–1.40); 0.392

Multivariable regression analyses for OS (*n* = 2009), PFS (*n* = 1956) and LRFS (*n* = 2046)

*HR* Hazard ratio, 95% Confidence Interval, *p* value; *RSC* Reference Sarcoma Centers, *ref* reference value

#### PFS

The 3y-PFS rates for AYAs in RSC, and AYAs in non-RSC were 55.6% (95%CI 52.3–58.8), and 50.4% (95%CI 46.4–54.3), respectively (Fig. 2B). In univariable analysis, PFS in AYAs in RSC was significantly improved compared to AYAs in non-RSC (HR 0.86, 95%CI 0.76–0.99).

After adjustment, AYAs in RSC showed improved PFS compared with AYAs in non-RSC (HR 0.83, 95%CI 0.71–0.97) (Table 3). PFS was reduced in patients with *de novo* metastatic disease (HR 2.15, 95%CI 1.79–2.58), grade 3 tumors (HR 2.20, 95%CI 1.83–2.64), ungraded tumors (HR 1.88, 95%CI 1.42–2.49). PFS was improved in patients with lower limb tumors (HR 0.76, 95%CI 0.66–0.89), and in patients with smaller tumor size (HR 0.79 95%CI 0.67–0.93) (Table 3).

#### OS

The 3y-OS rates for AYAs in RSC, and AYAs in non-RSC were 81.1% (95%CI 78.3–83.6) and 82.7% (95%CI 79.4–85.5), respectively (Fig. 2C). The univariable analysis identified no difference in OS between groups. After adjustment, we observed no significant differences in OS between AYAs in RSC and in non-RSC (HR 1.10, 95%CI 0.87–1.40). The OS was reduced in *de novo* metastatic disease (HR 2.90, 95%CI 2.29–3.67), grade 3 tumors (HR 3.27, 95%CI 2.11–5.05) (Table 3).

## Discussion

The present work used the French nationwide prospective database NETSARC to assess the survival of young patients 15–30 years with sarcoma diagnosed between 2010 and 2017. To the best of our knowledge, this study is the 1st to report the survival of young (15–30 years) patients treated for sarcoma at a national level. The present study reported 3y-OS rates in AYA patients with sarcoma of almost 80% which is consistent with the 2y-OS of 80% reported by Raze et al. in 2016 [4]. The study did not identify significant differences in OS between AYAs treated in RSC and in non-RSC, but being treated in RSC is associated with improved 3 y-LRFS and PFS. Of note, the present study considered in the AYA population all patients with age ranges from 15 to 30 years i.e. extended range compared to the usual 15–24 years as defined by *French National Cancer Institute* (INCa). Indeed, epidemiology based on advanced biological and histopathological characterisation of AYA neoplasms [21, 22], and sarcoma incidence related to pediatric histology (which stays significant up to the age of 30 years) both support the rationale for considering young adults 25–30 years in the group of AYAs [4, 23]. In addition, sarcoma patients aged from 25 to 30 years are still often managed in non-expert centers at the present time, and careful review by MDTB involving both pediatric and adult oncologists would also be advisable in these young adult population [12, 16, 24–26].

The initial management of patients with sarcoma is a highly sensitive issue. Reference centers showed better compliance to international guidelines and notably performed pathological review to confirm the absence of microscopically residual disease (R0) [5–7, 27, 28]. According to our results, the initial management in non-RSC showed less compliance with clinical practice guidelines with less pre-treatment procedures, including biopsy and imaging reported in only 48.1% and 56.5% of the patients, respectively. In addition, whereas the quality of first surgery in RSC is consistent with previous results (60% R0, 20% R1 and 5% R2) [9], the quality of surgery in non-RSC revealed only 20% R0 and up to 20% R2 although patients presented less negative prognostic criteria. In addition, the substantial reoperation rate may result from inadequate and/or inappropriate initial surgeries, and potential correlation with the lack of pre-treatment biopsy and imaging can be raised. Indeed, second resection after first macroscopic residual resection (R2) occurred in 44% of the AYAs first operated in RSC, and in up to 64% of the AYAs first operated in non-RSC. Among second resections performed after incomplete initial resection, re-operations were most often performed in expert centers (AYAs in RSC: 70%; AYA in non-RSC: 48%). These results support early referral to expert centers for initial surgery, and confirm results previously reported [9]. Whereas reoperations are mostly performed in RSC after first resection in non-RSC, few AYAs (*n* = 5) with first surgery in RSC were reoperated for incomplete resection in a non-expert center in this series. Nevertheless, such situations remain marginal and limited, and voluntary patient transition to non-RSC treatment centers for personal reasons cannot be excluded.

The quality of surgery is known as a major prognostic factor for relapse-free survival and overall survival in bone and soft tissue sarcomas [29–34]. However, in France as in the majority of European countries so far, the initial management of any sarcoma patient may be carried out in non-oncologic-specialized clinic, regardless of sarcoma expertise or number of sarcoma patients treated. Conversely, in the Scandinavian countries and the United Kingdom, a patient with sarcoma must be managed, upon suspicion, in an expert center [28, 35]. Resection in one of the 26 national expert centers reduced the risk of relapse by almost 35% in 35,784 sarcoma patients compared to those in non-expert centers [9]. Even if the difference is less clear than previously reported, our series showed that 3y-LRFS rates were 82% in AYAs managed in RSC compared to 71% in non-RSC. In addition, expert centers provide high-quality medical management including surgery complementary treatments. The highest level of expertise of the treatment center has to be required in young patients with sarcoma, and same requirements should also apply to the total young patient population < 30 years [14] to ensure accurate management and facilitate access to clinical trials [36]. This issue of particular concern prompted the *French National Cancer institute* INCa to create in 2011 the French academic society for AYA *Adolescent and Young Adult Oncology and Hematology Group* (GO-AJA) gathering pediatric and adult oncologists and hematologists at the national level [24]. However, at local level, patient management remains heterogenous, and young patients aged from 15 to 24 years may still be treated in any unit with expertise in oncology, with specific accreditation requirements for pediatric oncology to treat patients aged 15–18 years [25].

PFS and LRFS after adjustment for negative prognostic criteria were better in AYA patients treated in RSC compared to AYA managed in non-RSC (HR 0.83 and HR 0.58), respectively. Differences in PFS, and especially in LRFS, may result from a lower compliance with clinical practice guidelines at initial surgery, putting patients at risk for more frequent local and distant relapses. In addition, patients with higher grade tumors had worse PFS (HR 1.88) and LRFS (HR 1.82) compared to patients with grade 1–2 tumors which may reflect the increased aggressiveness of the disease both locally and at distant sites.

The absence of differences in OS may result from the current relatively short-term follow-up; a median follow-up of 39 months may be still insufficient to detect a significant difference in OS considering the 2y-OS of 80% and 5y-OS of 60% in AYA patients with sarcoma, aged 15 to 24 years in France [4]. Moreover, the influence of other non-observed potential negative prognosis factors in NETSARC centers cannot be excluded. Differences were evidenced in AYA characteristics in RSC and in non-RSC; we reported more bone sarcomas, lower limb tumors, large tumors, and grade 3 tumors in AYAs in RSC. Conversely, AYAs in non-RSC had more STS, non-grade 3 tumors, and smaller tumor sizes. This heterogeneity in patient characteristics may lead to the observed patient referral in France: patients presenting with negative prognostic criteria (grade 3, larger size, and metastatic status at diagnosis) are easily diagnosed and therefore referred to an expert center. As a consequence, a majority of bone sarcomas and lower limb tumors are managed in expert centers. The first actor in the patient management is most often an orthopaedic surgeon, which may be reluctant for resection of bone tumor presenting negative clinical or paraclinical prognostic criteria. Thus, expert centers are more likely to be consulted for diagnosis and therapeutic advice. For patients presenting with small /superficial STS is more likely to be directed to digestive or plastic surgeon in non-expert sarcoma centers, more often confronted with benign STS, and decision for resection adopted without resorting to further expertise, neither pre-surgery review for more accurate diagnostics.

The AYA population in this study showed more STS (60%) than bone sarcomas (40%), which contrasts with the rates previously reported by Raze and colleagues in 2018 in the 15–24 years old patients (bone sarcoma: 53%; STS: 47%) [4]. This shift to increased STS rate in our overall AYA population may result from the STS/bone sarcoma ratio of 9/10 reported in adults, and therefore supported in our series by the 25–30 year old population [23].

### Limits of our study

The NETSARC database does not collect all medical treatments chemotherapy and targeted therapy administered before and after surgery, and do not allow to explore the impact of medical treatments administered in RSC and non-RSC so far. However, from 2016, additional data collection showed that neoadjuvant treatment administration was mostly observed in AYA in RSC (56.9%) and rarely in AYA patients in non-RSC (14%), which may have contributed to better local control and to increase 3y-LRFS in AYA in RSC. Detailed information regarding neoadjuvant treatment (systemic anticancer drugs, radiotherapy or combination thereof) are not specified and impact on 3y-PFS in AYA in RSC are not accessible.

Comparisons in sarcoma management in AYAs are a critical issue not only regarding age ranges but also sarcoma histotype representations. If embryonal tumors are obviously more common in children than in AYAs, representation of sarcoma histotypes in AYA is closer to pediatric than to adult sarcomas. Whereas pediatric sarcoma treatment is exclusively performed in reference centers in France, and showed a quality of management recognized as a robust reference, all the AYAs up to 30 years may also derive benefit from a similar high-quality management. Subgroup analyses according to histotype would be highly informative, but have not been achieved so far, still limited by the high heterogeneity of sarcomas.

## Conclusions

In France, AYA patients with sarcoma managed in an expert center showed better compliance to guidelines at diagnosis and improved LRFS and PFS. With the current median follow-up of 39 months, no significant difference in 3y-OS was observed in our series. The present study outlines the importance of earlier access to reference sarcoma centers with multidisciplinary MDTBs, involving pediatric and adult oncologists, for any patient aged 15–30 years with suspicion of sarcoma.

## Data Availability

The nationwide database NETSARC (https://netsarc.sarcomabcb.org) that support the findings of this study contains information that could compromise privacy of the research participants. Anonymised data sets are available upon reasonable request from the data protection officer of the Léon Bérard cancer center at DPD@lyon.unicancer.fr.
